# CircKEAP1 Suppresses the Progression of Lung Adenocarcinoma *via* the miR-141-3p/KEAP1/NRF2 Axis

**DOI:** 10.3389/fonc.2021.672586

**Published:** 2021-05-31

**Authors:** Yanbo Wang, Fenghai Ren, Dawei Sun, Jing Liu, BenKun Liu, YunLong He, Sainan Pang, BoWen Shi, FuCheng Zhou, Lei Yao, YaoGuo Lang, ShiDong Xu, JunFeng Wang

**Affiliations:** ^1^ Department of Thoracic Surgery, Harbin Medical University Cancer Hospital, Harbin, China; ^2^ Department of Gastroenterology and Hepatology, The 2nd Affiliated Hospital of Harbin Medical University, Harbin, China

**Keywords:** circRNA, lung adenocarcinoma, circKEAP1, KEAP1, cell proliferation

## Abstract

**Background:**

Lung cancer is the leading cause of death from cancer, and lung adenocarcinoma (LUAD) is the most common form. Despite the great advances that has been made in the diagnosis and treatment for LUAD, the pathogenesis of LUAD remains unclear. In this study, we aimed to identify the function of circKEAP1 derived from the exon of KEAP1 in LUAD.

**Methods:**

The expression profiles of circRNAs in LUAD tissues and adjacent non-tumor tissues were analyzed by Agilent Arraystar Human CircRNA microarray. The levels and prognostic values of circKEAP1 in tissues and cancer cell lines were determined by quantitative real-time PCR (qRT-PCR). Subsequently, the effects of circKEAP1 on tumor growth were investigated by functional experiments *in vitro* and *in vivo*. Mechanistically, the dual luciferase reporter assay, RNA pull-down, and RNA immunoprecipitation experiments were performed to confirm the interaction between circKEAP1 and miR-141-3p in LUAD.

**Results:**

We found circKEAP1 was significantly downregulated in LUAD tissues and repressed tumor growth both *in vitro* and *in vivo*. Mechanistically, circKEAP1 competitively binds to miR-141-3p and relive miR-141-3p repression for its host gene, which activated the KEAP1/NRF2 signal pathway, and finally suppresses the tumor progress. Our findings suggest that circKEAP1 inhibits LUAD progression through circKEAP1/miR-141-3p/KEAP1 axis and it may serve as a novel method for the treatment of LUAD.

## Introduction

Lung cancer is the leading cause of cancer-related death worldwide (1). Lung adenocarcinoma (LUAD) is the most common histological class of lung cancer, approximately 60%. Despite the great development of treatment for some genetic subtypes of human LUAD, the overall 5-year survival of LUAD remains less than 20% (2). Moreover, the morbidity of LUAD is continuously raised during the past years, especially in women, never-smokers, and young adults (1). Therefore, it is urgent to identify the molecular mechanisms of LUAD to develop new therapeutic methods.

Circular RNAs (circRNAs) are new endogenous RNAs that could resistant to RNase R degradation due to their covalent closed-loop structure (3). In the past decades, circRNAs were thought to be the product of splicing errors. Recently, several studies have found that circRNAs can act as miRNA sponges, protein sponges, transporters, or scaffolds, which in turn are involved in the development and progression of several diseases, especially cancer (3, 4). With the development of high-throughput detection of circRNA technologies, such as microarray and RNA-seq, more and more studies have found the expression profiles of circRNAs in tumor tissues and paracancerous tissues have significant differences. CircRNAs in tumor cells may play an important role in tumor development by affecting processes, such as tumor cell proliferation, epithelial-to-mesenchymal transition (EMT), angiogenesis, and apoptosis (5). circRNAs have become a potential target for tumor therapy. In addition, due to the stability of circRNAs, circRNAs are also considered as a novel kind of promising biomarker for tumor diagnosis, prognosis, and individualized therapy. The major function of circRNAs is regarded as miRNA sponges (6). MiRNAs are novel ~22nt non-coding RNAs that repress gene expression (7). It is known that the downregulation of some tumor suppressor genes in cancer may be caused by an increase in miRNAs (known as oncomiR), while a decrease in tumor suppressor miRNAs (known as tumor suppressor miRNAs) leads to an upregulation of oncogenes (7). Most of the circRNAs contain miRNA binding sites and could inhibit the regulation of miRNAs on their target gene mRNAs, thereby regulating target gene expression at the post-transcriptional level (3). In LUAD, several circRNAs have been revealed to be significantly dysregulated and could act as ceRNA to sponge oncomiR or tumor suppressor miRNAs (8–13). For example, we found has-circRNA-002178 were significantly upregulated in the LUAD and could enhance PDL1 expression *via* sponging miR-34 in cancer cells to induce T-cell exhaustion (14).

In our study, the expression profile of circRNAs in LUAD was explored by circRNA microarray. We found a novel LUAD-related circRNA, named circKEAP1, was significantly downregulated in lung adenocarcinoma tissues. Subsequently, circKEAP1 was confirmed to act as a sponge of mir-141-3p, relieve the inhibition of miR-141-3p on its target gene KEAP1, and activate KEAP1/NRF2 signaling pathway, thereby inhibiting cancer cell proliferation and migration. In summary, our data show that circKEAP1 might act as a tumor suppressor *via* miR-141-3p-KEAP1-NRF2 axis in LUAD.

## Materials and Methods

### Patients’ Characteristics

CircRNA and miRNA expression profiles for seven paired LUAD cancer tissues and adjacent normal tissues were generated using the Human CircRNA microarray and Human miRNA Expression Assay. The clinic pathological features of these seven patients are described in **Supplementary Table S1**. We collected 105 cancer tissues and paired distal normal tissues from patients who were firstly diagnosed with LUAD without any treatment at the Harbin Medical University Cancer Hospital (Harbin, China). The clinic pathological features are described in **Supplementary Table S2**. The ethics committee of the Harbin Medical University Cancer Hospital authorized the study, and we conducted it in conformity to the Declaration of Helsinki.

### Cell Culture

Normal human bronchial epithelial cell line BEAS-2B, human LUAD cell lines A549 and PC9 was purchased from American Type Culture Collection (ATCC) (Manassas, VA, USA). A549 and PC9 were cultured in DMEM Medium (Gibco, Carlsbad, CA, USA) with 10% FBS, 10,000 units/ml penicillin, and 10,000 μg/ml streptomycin. BEAS-2B was cultured in RPMI 1640 Medium (Gibco, Carlsbad, CA, USA) with 10% FBS, 10,000 units/ml penicillin, and 10,000 μg/ml streptomycin. All the cells were maintained in a 5% CO_2_ humidified atmosphere at 37°C.

### RNA/gDNA Extraction and RT-PCR/qRT-PCR Assay

Total RNAs were isolated by TRIzol reagent (Takara, Dalian, China) according to the manufacturer’s instruction. gDNA was isolated by Genomic DNA Isolation Kit (Sangon Biotech, Shanghai, China). The quality and quantity of RNA and DNA were detected by Nanodrop 2000 spectrophotometer (Thermo Fisher Scientific, USA). The nuclear and cytoplasmic fractions were purified by PARIS Kit (Ambion, Life Technologies). RNA was reverse transcribed by HiScript II Q RT SuperMixfor qPCR (+gDNA wiper) (Vazyme, Nanjing, China). The AmpliTaq DNA Polymerase (Life Technologies) was used for PCR. The 2% agarose gel electrophoresis was performed to observe the cDNA and gDNA PCR products. AceQ qPCR SYBR Green Master Mix (Vazyme, Nanjing, China) was used for qRT-PCR, and GAPDH was used to normalize the level of circRNA and mRNA. Hydrolysis probe-based RT-qPCR assay of miRNA was performed according to the manufacturer’s instructions (Applied Biosystems). The miRNA level was normalized by small nuclear U6. Primers are listed in **Supplementary Table S3**.

### RNase R Treatment

Total RNA (about 2 mg) was incubated with 5 U/μg RNase R (Epicentre Technologies) for 30 min at 37°C. Then, the RNA was purified by RNeasy MinElute Cleaning Kit (Qiagen) according to the manufacturer’s protocols.

### Actinomycin D Assay

BEAS-2B cells were exposed to 2 μg/ml actinomycin D (Sigma) at indicated time point. Subsequently, the cells were harvested to extract the total RNA, and the stability of circKEAP1 and KEAP1 mRNA was analyzed using qRT-PCR.

### Vector Construction and Cell Transfection

To overexpress circKEAP1, the full-length cDNA of circR-KEAP1 was synthesized and then cloned into pLCDH-ciR plasmid (Geneseed, Guangzhou, China). For luciferase reporter vector, the sequence of circKEAP1, mutated circKEAP1 (the seed sequence mutated from UGGUGUU to ACCACAA for binding site 1, and CAGCGUUG to GUCGCAAC for binding site 2), KEAP1 3'UTR and mutated KEAP1 3'UTR (the seed sequence mutated from CAGUGUU to GUCACAA) was synthesized and then cloned into the pGL3-promoter vector (Geneseed, Guangzhou, China). The Dual Luciferase Assay Kit (Promega, Madison, WI, USA) was used to examine the luciferase activity accordance to the manufacturer’s protocols. SiRNAs of circKEAP1, miRNA mimics, miRNA inhibitors, and corresponding negative control (NC) were synthesized by GenePharma (Shanghai, China). Cells were transfected using Lipofectamine 3000 (Invitrogen) according to the manufacturer’s instruction.

### Cell Proliferation Assays

The proliferation activity of A549 and PC9 cells was tested by both Cell-Light™ EdU DNA Cell Proliferation Kit (Ribobio, Guangzhou, China) and Cell Counting Kit-8 (Dojindo Laboratories, Kumamoto, Japan) following the manufacturer’s protocols.

### Cell Migration Assays

The cell migration was detected by the Transwell chamber (Corning Life Sciences, Corning, NY, USA). Firstly, 5 × 10^4^ cancer cells was fixed with serum-free DMEM (Thermo Fisher Scientific) and seeded into the upper chamber, and the DMEM containing 20% serum was put into the lower chamber. After incubation for 24 h, the cells in lower surface of the upper chamber were fixed by 4% formaldehyde solution for 2.5 h, and then stained with crystal violet (Corning Life Sciences). After thoroughly washed, the migrated cells were observed by the microscope.

### MTT Assays

The cell viability of cancer cells was evaluated by the MTT Cell Proliferation Assay Kit (Beyotime, Shanghai, China). Cells were seeded in 96-well plate (10^4^/per well) and were incubated with MTT solution (10 μl, 2 h at 37°C) at 12, 24, 36, 48 h. The absorbance was detected by a spectrometer reader (Olympus, Tokyo, Japan).

### RNA Immunoprecipitation (RIP) and Biotin-Coupled miRNA Capture

The Magna RIP RNA-Binding Protein Immunoprecipitation Kit (Millipore, Billerica, MA) was used for RIP experiments according to the manufacturer’s instructions. AGO2 antibody used for RIP was purchased from Cell Signaling Technology. The 3' end biotinylated miR-141-3p mimics or control RNA (Ribio, Guangzhou, China) were transfected into BEAS-2B cells for 48 h before harvest. Then, we added cell lysis buffer [5 mM MgCl_2_, 100 mM KCl, 20 mM Tris (pH 7.5), 0.3% NP-40, 50 U of RNase OUT (Invitrogen, USA)] and complete protease inhibitor cocktail (Roche Applied Science, IN) into the cell pellets, and incubated them on ice for 30 min. The biotin-coupled RNA complex was pulled down by incubating the cell lysates with streptavidin-coated magnetic beads (Life Technologies) by centrifugation at 10,000*g* for 20 min.

### Western Blot Analysis

The protein extraction reagent (Thermo Scientific) with a cocktail of proteinase inhibitors (Roche Applied Science, Switzerland) was used to isolate the total protein from cells or tissue samples. Equal amount of total protein was separated by 10% SDS-PAGE and transferred onto a PVDF membrane. Then, the membranes were blocked with 5% skimmed milk powder and incubated the membranes with primary antibodies at 4°C overnight and then incubated with secondary antibodies at room temperature for 2 h. The bands were examined by Immobilob™ Western Chemiluminescent HRP Substrate (Millipore, Billerica, MA, USA). The primary antibody and secondary antibodies were purchased from Cell Signaling Technology, and the detailed information list below: KEAP1 [KEAP1 (D6B12) Rabbit mAb #8047; Cell Signaling Technology, Beverly, MA, USA], GAPDH [GAPDH (D16H11) XP^®^ Rabbit mAb #5174; Cell Signaling Technology], NRF2 [NRF2 (D1Z9C) XP^®^ Rabbit mAb #12721; Cell Signaling Technology], HDAC4 [HDAC4 (D8T3Q) Rabbit mAb #15164, Cell Signaling Technology], and the secondary antibodies (Anti-rabbit IgG, HRP-linked Antibody #7074; Cell Signaling Technology).

### Animal Experiments

Stably over-expressed cell lines were established by transfecting A549 and PC9 cells with *circKEAP1* overexpressed plasmid or control plasmid and selected with puromycin. Then, we suspended 10^6^ A549 cells in 100 μl PBS and injected into the right flank of 6-week-old male BALB/c nude mice. We measured the volume of tumors every 5 days. After 20 days, the mice were sacrificed and the tumors were collected for further analysis.

### Statistical Analyses

Adequate sample size was determined according to the previous studies that performed analogous experiments. A two-sided test was applied. The raw data applying *t* test was normally distributed. Data are expressed throughout the manuscript as mean ± SD. The SPSS 18.0 software was performed to the statistical analyses, and the GraphPad Prism 6.0 (GraphPad Software, San Diego, CA, USA) was used to generate the graphs. A P-value <0.0.05 was regarded as statistically significant.

## Results

### CircKEAP1 Is Downregulated in LUAD

**Figure 1:**
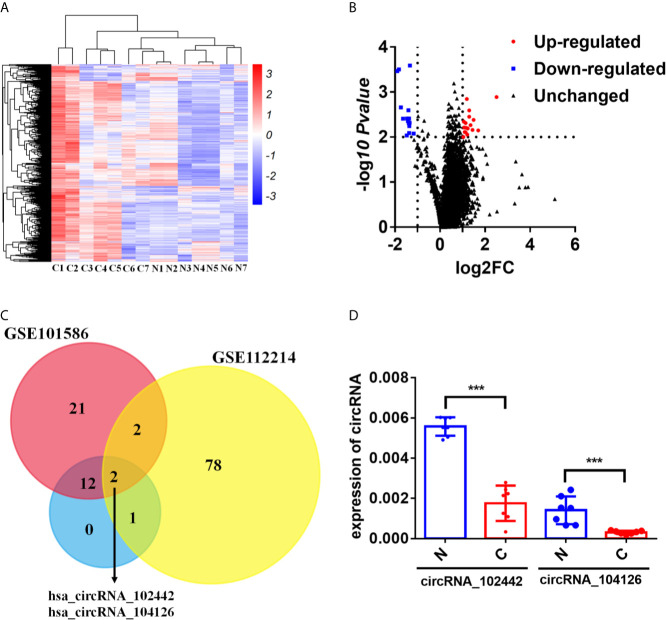
circKEAP1 was significantly downregulated in LUAD by circRNA microarray. **(A, B)** The heatmap **(A)** and volcano plot **(B)** of circRNA profiles in lung adenocarcinoma tumor tissues (C) and adjacent normal tissues (N). **(C)** Venn diagrams presenting circRNAs downregulated in our circRNA expression profile and circRNA expression profile of GSE112214 and GSE101586. **(D)** qRT-PCR for the abundance of hsa_circRNA_104126 and hsa_circRNA_102442 in the seven paired lung adenocarcinoma tumor tissues (C) and adjacent normal tissues (N). Data are shown as the means ± standard error of the mean (n = 3), statistical analysis was performed by two-tailed Student’s t test; ***P < 0.001.

The human CircRNA microarray was explored to analyze the expression profile of circRNAs in seven paired lung adenocarcinoma tissues and adjacent normal tissues. 32 dysregulated circRNAs were found (fold change>2 and *p*-value < 0.05). Compared to the adjacent normal tissues, 15 circRNAs were downregulated and 17 circRNAs were upregulated in lung adenocarcinoma tissues (**Figures 1A, B** and **Supplementary Table S4**). To further analyze the dysregulated circRNAs in LUAD, another two circRNA expression profiles (GSE112214 and GSE101586) of LUAD in GEO database were investigated. The results showed that hsa_circRNA_104126 and hsa_circRNA_102442 were significantly downregulated in all the three circRNA expression profiles (**Figure 1C**). Then, the expression level of hsa_circRNA_104126 and hsa_circRNA_102442 was confirmed in the seven paired samples for microarray analysis by qRT-PCR. Consistent with the results of the circRNA expression profiles, hsa_circRNA_104126 and hsa_circRNA_102442 were remarkably downregulated in the cancer tissues (**Figure 1D**). Since the expression level and fold change of hsa_circRNA_102442 was much higher than hsa_circRNA_104126 (**Figure 1D**), hsa_circRNA_102442 was chosen for further study.

**Figure 2:**
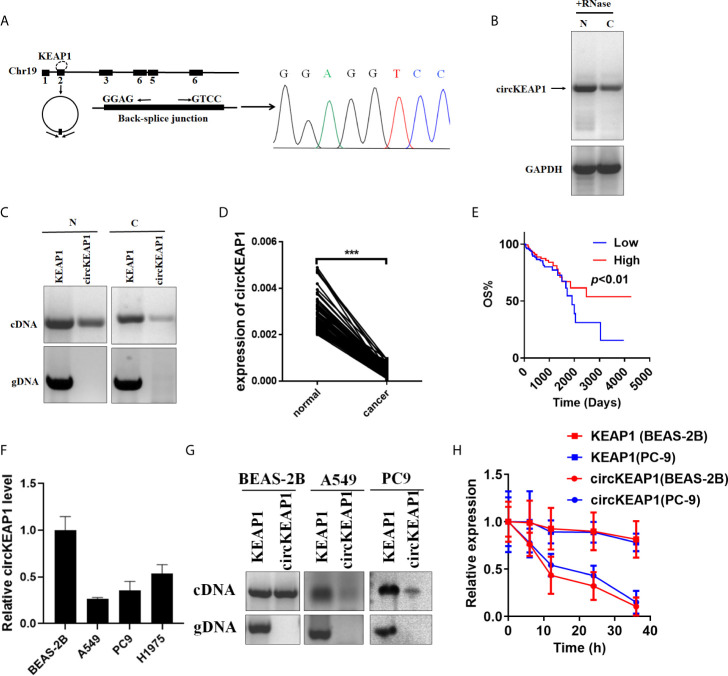
Characterization of circKEAP1 in LUAD. **(A)** Genomic loci of circKEAP1 gene. CircKEAP1 is produced at the KEAP1 gene locus containing exon 2. The back-splice junction of circKEAP1 was identified by Sanger sequencing. **(B)** Northern blot analysis showed the abundance of circKEAP1 in one paired sample of LUAD cancer tissue (C) and adjacent normal tissues (N). **(C)** PCR analysis for circKEAP1 and its linear isoform KEAP1 in cDNA and genomic DNA (gDNA) in one paired sample of LUAD cancer tissue (N) and adjacent normal tissues (C). **(D)** qRT-PCR for the abundance of circKEAP1 in 105 paired samples of LUAD cancer tissues (cancer) and adjacent normal tissues (normal). **(E)** The association of circKEAP1 expression with the overall survival of LUAD patients was analyzed by Kaplan-Meier method. Log-rank test. **(F)** qRT-PCR revealed the expression of circKEAP1 in LUAD cells (A549, PC9, H1975) relative to normal cells (BEAS-2B). **(G)** PCR analysis for circKEAP1 and its linear isoform KEAP1 in cDNA and genomic DNA (gDNA) in human LUAD cell lines A549 and PC9, and normal human bronchial epithelial cell line BEAS-2B. **(H)** qRT-PCR for the abundance of circKEAP1 and KEAP1 in BEAS-2B and PC9 cells treated with actinomycin D at the indicated time point. Data are shown as the means ± standard error of the mean (n = 3), statistical analysis was performed by two-tailed Student’s *t* test; ***P < 0.001.

Hsa_circRNA_102442 (named circKEAP1) is located at chr19: 10610070-10610756, and derived from the second exon of the KEAP1 gene with a length of 686nt by alternative splicing (**Figure 2A**). The circKEAP1 was confirmed by Sanger sequencing through the PCR which amplified the back-spliced junction of circKEAP1 by divergent primers (**Figure 2A**). The Northern blot analysis was performed by a probe that targeted the back-spliced junction, and the result showed that circKEAP1 could be observed at 686 nt in the lung tissues (**Figure 2B**). Additionally, PCR analysis for reverse-transcribed RNA (cDNA) and genomic DNA (gDNA) showed that divergent primers could amplify products from cDNA of tissues but not from gDNA (**Figure 2C**). Subsequently, the expression level of circKEAP1 in 105 paired cancer tissues and adjacent normal tissues from LUAD patients was analyzed by quantitative reverse transcription PCR (qRT-PCR). As shown in **Figure 2D**, the expression of circKEAP1 was obviously decreased in cancer tissues (**Figure 2D**). Kaplan-Meier analysis implied that low circKEAP1 was associated with unfavorable prognosis in LUAD patients (**Figure 2E**). Moreover, we also found that low circKEAP1 expression was closely associated with lymphatic metastasis and tumor stage (**Supplementary Table S5**).

Next, the expression of circKEAP1 was determined in lung adenocarcinoma cell lines (A549, PC9, H1975) and normal lung epithelial cell line (BEAS-2B). Results showed that circKEAP1 level was reduced in lung adenocarcinoma cell lines (**Figure 2F**). Gel electrophoresis exposed that circKEAP1 was amplified by divergent primers only from cDNA instead of gDNA in BEAS-2B cells (**Figure 2G**). Subsequently, the BEAS-2B and PC9 cells were treated with Actinomycin D (an inhibitor of transcription) to explore the stability of circKEAP1 and the KEAP1 mRNA. As shown in **Figure 2H**, the half-life of circKEAP1 transcript exceeded 24 h and was much longer than the KEAP1 mRNA.

### circKEAP1 Inhibits Tumor Growth

**Figure 3:**
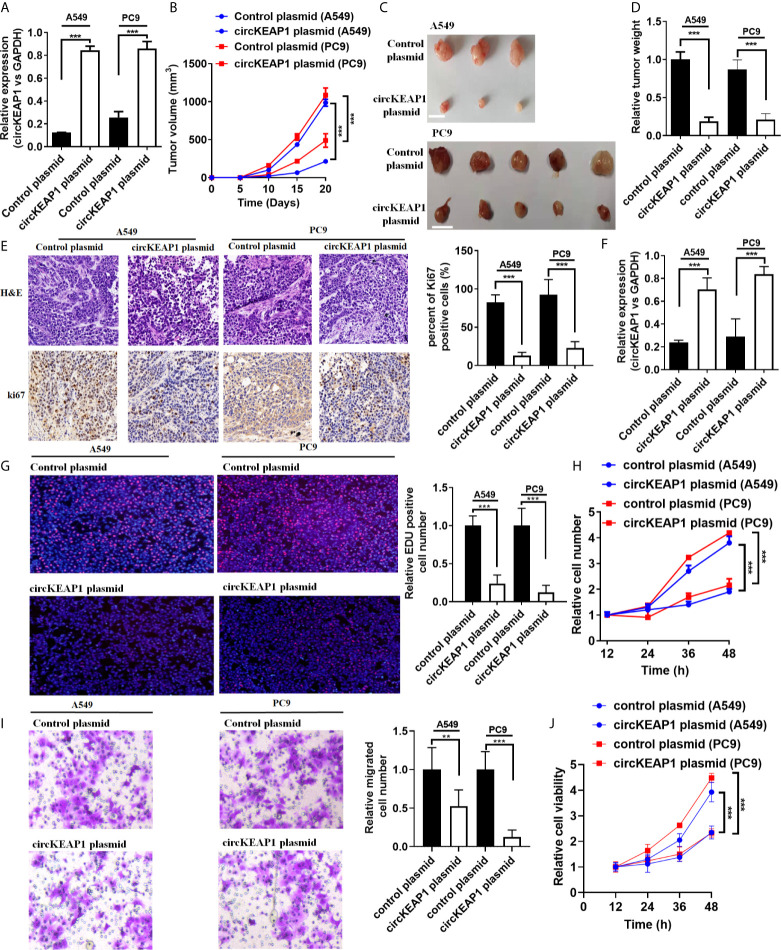
circKEAP1 inhibits tumor growth. **(A)** The expression level of circKEAP1 in A549 and PC9 cells transfected with control plasmid or circKEAP1 plasmid. **(B)** The volume of subcutaneous xenograft tumors of A549 and PC9 cells isolated from nude mice. **(C, D)** The weight of subcutaneous xenograft tumors of A549 and PC9 cells isolated from nude mice. The graduated scale is 1 cm. **(E)** HE staining and IHC staining for Ki-67 in xenografted tumors. **(F)** Expression levels of circKEAP1 in xenografted tumors. **(G)** Cell proliferation analysis for A549 and PC9 cells transfected with control plasmid or circKEAP1 overexpression plasmid by EDU assay. **(H)** Cell proliferation analysis for A549 and PC9 cells transfected with control plasmid or circKEAP1 overexpression plasmid by CCK-8 assay. **(I)** Cell migration analysis for A549 and PC9 cells transfected with control plasmid or circKEAP1 overexpression plasmid by Transwell assay. **(J)** Cell viability analysis for A549 and PC9 cells transfected with control plasmid or circKEAP1 overexpression plasmid by MTT assay. Data are shown as the means ± standard error of the mean (n = 3), statistical analysis was performed by two-tailed Student’s t-test; **P < 0.01, ***P < 0.001.

To study the role of circKEAP1 in LUAD progression, the full-length cDNA of circKEAP1 was synthesized and cloned into the pLCDH-ciR plasmid to generate the overexpression plasmid of circKEAP1 (circKEAP1 plasmid), which contained a front and back circular frame. The overexpression plasmid of circKEAP1 successfully increased the expression level of circKEAP1 in A549 and PC9 cells (**Figure 3A**). A nude mice xenograft model by implanting A549 and PC9 cells transfected with control plasmid or circKEAP1 plasmid was established to identify the effect of circKEAP1 on tumor growth *in vivo*. The A549 and PC9 cells were subcutaneously injected into the right flank of 6-week-old male BALB/c nude mice. The tumor volumes were monitored from the 5 days after A549 and PC9 cell injection, and the mice were sacrificed after 20 days. The results showed overexpression of circKEAP1 drastically suppress the tumor growth of both A549 and PC9 cells (**Figures 3B, C**). The tumor weights were remarkably decreased by the overexpression of circKEAP1 (**Figure 3D**). As shown in **Figure 3E**, both H&E staining and Ki-67 staining of these tumors showed decreased cell mitosis and a lower percentage of proliferative cells in the circKEAP1 overexpression group. As the previous results in the A549 and PC9 cells (**Figure 3A**), the expression level of circKEAP1 was much higher in the circKEAP1 overexpression group, compared to the control group (**Figure 3F**). These data indicated that circKEAP1 repressed tumor growth *in vivo*.

To identify the function of circKEAP1 on lung adenocarcinoma cancer cells *in vitro*, the EDU, CCK-8, Transwell, and MTT assay were performed to the A549 and PC9 cells transfected with control plasmid or circKEAP1 plasmid. As the results shown in **Figures 3G–J**, circKEAP1 significantly suppressed the cancer cell proliferation and migration *in vivo*.

In summary, these findings suggest that circKEAP1 could repress the tumor growth.

### circKEAP1 Act as a Sponge for miR-141-3p

**Figure 4:**
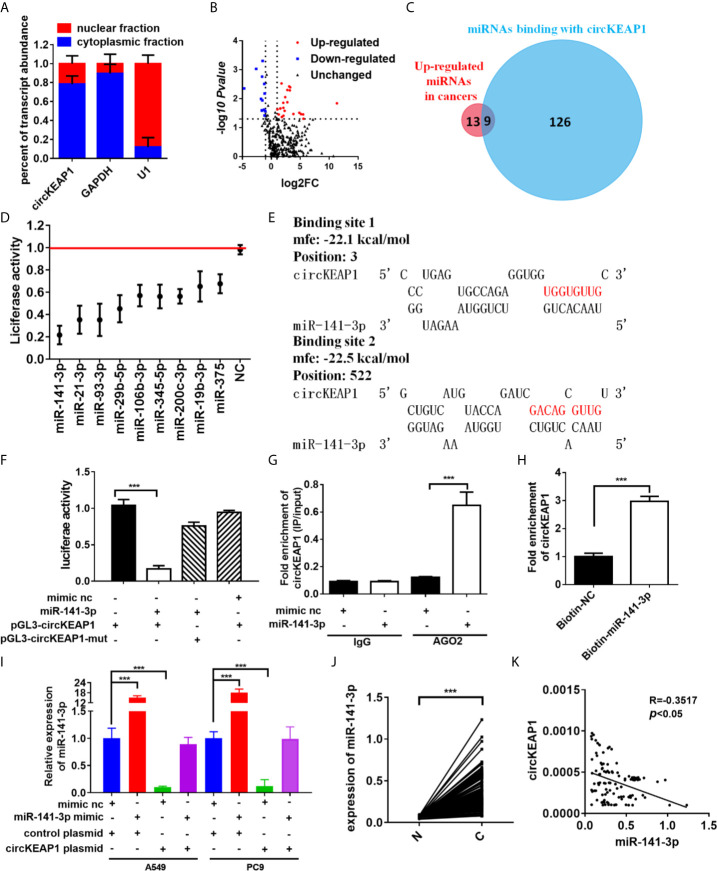
circKEAP1 acts as a sponge for miR-141-3p. **(A)** Levels of circKEAP1 in the nuclear and cytoplasmic fractions of BEAS-2B cells. **(B)** The volcano plot of miRNA in seven paired lung adenocarcinoma tumor tissues and adjacent normal tissues. **(C)** A schematic model shows the putative binding sites of nine predicted miRNAs on circKEAP1. **(D)** Luciferase activity of circKEAP1 in A549 cells transfected with miRNA mimics which are putative binding to the circKEAP1 sequence. Luciferase activity was normalized by Renila luciferase activity. **(E)** The binding sites of miR-141-3p with circKEAP1 were predicated *via* targetScan. **(F)** Luciferase reporter activity of circKEAP1 in A549 cells co-transfected with miR-141-3p mimics and circKEAP1 luciferase reporter plasmid. **(G)** RIP was performed using AGO2 antibody in BEAS-2B cells transfected with miR-141-3p mimics or mimics NC, then the enrichment of circKEAP1 was detected. **(H)** circKEAP1 was pulled down and enriched with 3'-end biotinylated miR-141-3p in BEAS-2B cells. **(I)** Expression levels of miR-141-3p in A549 and PC9 cells co-transfected with miR-141-3p mimics and circKEAP1 overexpression plasmid. **(J)** qRT-PCR for the abundance of miR-141-3p in 105 paired LUAD cancer tissues (C) and adjacent normal tissues (N). **(K)** Pearson analysis the relationship between miR-141-3p and circKEAP1 in expression in LUAD tissues **(K)**. Data are shown as the means ± standard error of the mean (n = 3), statistical analysis was performed by two-tailed Student’s *t* test; ***P < 0.001.

In order to identify the mechanism that circKEAP1 suppressed the progression of lung adenocarcinoma, we firstly observed the cellular localization of circKEAP1 by qRT-PCR. As shown in **Figure 4A**, the circKEAP1 transcript was preferentially located in the cytoplasm. Most of the circRNAs in the cytoplasm are reported to be involved in cancer by competing with miRNAs (3, 4). CircKEAP1 was speculated to be severed as a miRNA sponge. To test the hypothesis, the miRNA expression profile of the seven paired LUAD cancer tissues and non-tumor tissues was analyzed by Human miRNA Expression Assay. The volcano map showed that the miRNAs profiles were significantly different between the lung adenocarcinoma tumor tissues and the adjacent normal tissues (**Figure 4B** and **Supplementary Table S6**). Then, the potential binding sites of miRNAs in the circKEAP1 were predicted by Targetscan (15), 135 miRNAs were identified as potential targets for circKEAP1 (**Supplementary Table S7**). As the circKEAP1 downregulated in lung adenocarcinoma tumor tissues (**Figure 2D**), the predicted miRNA which was upregulated in the lung adenocarcinoma tumor tissues might be sponged by circKEAP1. The resulted showed nine miRNAs, including miR-141-3p, miR-106b-3p, miR-93-3p, miR-21-3p, miR-19b-3p, miR-29b-5p, miR-3445-5p, miR-375, and miR-200c-3p, contained at least one binding site with circKEAP1 and significantly upregulated in lung adenocarcinoma tumor tissues (**Figure 4C**). Then, a dual-luciferase reporter assay was performed to further confirm the interaction between circKEAP1 and the nine miRNAs in A549 cells. Among the nine predicted miRNAs, all the miRNAs significantly attenuated the luciferase activity of A549 cells, and the miR-141-3p was the most obvious and selected for deeper investigation (**Figure 4D**).

As shown in **Figure 4E**, there were two binding sites between circKEAP1 and miR-141-3p. Subsequently, the dual-luciferase reporter assay was performed to confirm the bioinformatics prediction analysis in A549 cells. The full length of circKEAP1 and muted circKEAP1 with muted miR-141-3p binding sites were cloned into luciferase reporter pGL3 plasmid, respectively. MiR-141-3p mimic effectively increased the expression level of miR-141-3p in A549 cells (Figure S1), and significantly decreased the luciferase activity of the wildtype group, but it had no effect on the mutant group (**Figure 4F**). These results suggested circKEAP1 could directly bind with miR-141-3p. CircRNAs have been proved to bind with miRNAs through AGO2 (Argonaute 2) (16). Therefore, the anti-AGO2 RNA immunoprecipitation (RIP) assay was conducted in BEAS-2B cells with anti-AGO2 antibody. Interestingly, both circKEAP1 and miR-141-3p were efficiently pulled down by anti-AGO2 antibodies (**Figure 4G**). Then, the miRNA pull-down assay with specific biotin-labeled miR-141-3p was performed to further verify the binding of circKEAP1 and miR-141-3p. As expected, circKEAP1was significantly enriched in the biotin-labeled miR-141-3p group compared with the control (**Figure 4H**). Additionally, we also found the expression level of miR-141-3p in both A549 and PC9 cells (**Figure 4I**) and tumors (**Supplementary Figure S2A**) was markedly decreased when we overexpressed the circKEAP1 by circKEAP1 plasmid. The decrease could be attenuated by miR-141-3p mimic (**Figure 4I**). Finally, the expression level of miR-141-3p in the 105 paired cancer tissues and adjacent non-cancerous tissues was examined. The results showed miR-141-3p was markedly upregulated in lung adenocarcinoma tumor tissues (**Figure 4J**) and had a negative correlation with circKEAP1 (**Figure 4K**).

These results revealed that circKEAP1 could serve as a sponge for miR-141-3p.

### miR-141-3p Repress the KEAP1 Expression in Tumors

**Figure 5:**
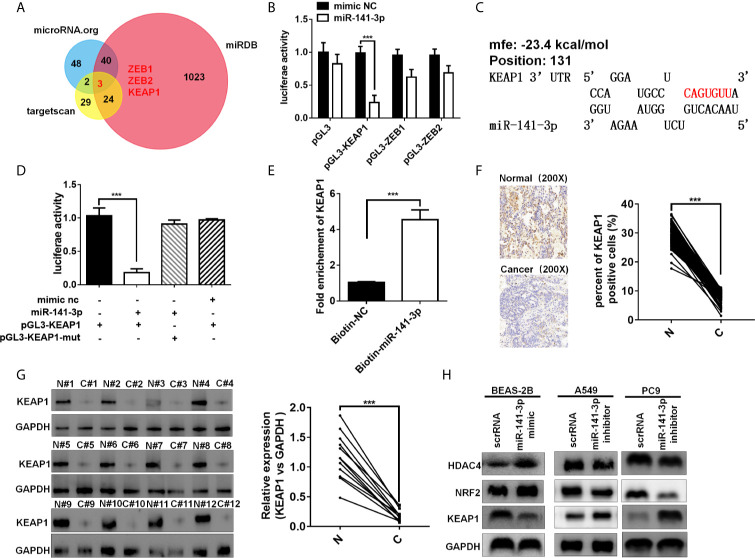
miR-141-3p inhibits KEAP1 expression. **(A)** Putative target genes of miR-141-3p were predicated by trargetScan, miRDB, and miRDana. **(B)** Luciferase activity of miR-141-3p in A549 cells transfected with luciferase reporter plasmid containing the putative binding of target genes to miR-141-3p. Luciferase activity was normalized by Renila luciferase activity. **(C)** The binding sites of miR-141-3p with KEAP1 were predicated *via* targetScan. **(D)** Luciferase reporter activity of KEAP1 in A549 cells co-transfected with miR-141-3p mimics and KEAP1 luciferase reporter plasmid. **(E)** KEAP1 was pulled down and enriched with 3'-end biotinylated miR-141-3p in BEAS-2B cells. **(F)** Expression levels of KEAP1 protein in 105 paired LUAD cancer tissues (C) and adjacent normal tissues (N) by IHC staining. **(G)** Expression levels of KEAP1 protein in 12 paired LUAD cancer tissues (C) and adjacent normal tissues (N) by western blotting. **(H)** The protein levels of KEAP1 in BEAS-2B cells transfected with mimics of miR-141-3p and A549 cells transfected with inhibitors of miR-141-3p. Data are shown as the means ± standard error of the mean (n = 3), statistical analysis was performed by two-tailed Student’s *t* test; ***P < 0.001.

Three bioinformatics software TargetScan (15), miRDB (17), and miRanda (18) were used to predict the target genes of miR-141-3p. Bioinformatics analysis showed that the 3′-UTRs of ZEB1, ZEB2, and KEAP1 contained miR-141-3p complementary sequences by the three algorithms (**Figure 5A**). The dual luciferase reporter assay showed that miR-141-3p remarkably suppressed the luciferase activity of luciferase reporter vector containing the *KEAP1* 3'UTR sequence, while it has no effect on ZEB1 and ZEB2 in A549 cells (**Figure 5B**). As predicted, miR-141-3p has one binding site in the 3'-UTR of KEAP1 (**Figure 5C**). In order to further confirm the repression of miR-141-3p on KEAP1, the luciferase reporter vector containing the *KEAP1* 3'UTR wild type sequence or the muted *KEAP1* 3'UTR sequence was generated. The results showed that miR-141-3p remarkably suppressed the luciferase activity of luciferase reporter vector containing the *KEAP1* 3'UTR wild type sequence, while it did not have influence on the muted vector containing the muted *KEAP1* 3'UTR sequence (**Figure 5D**). Subsequently, the miRNA pull-down assay was performed with specific biotin-labeled miR-141-3p, and the result showed that KEAP1 mRNA was enriched in the biotin-labeled miR-141-3p group, like circKEAP1 (**Figure 5E**). The immunohistochemistry for KEAP1 protein in 105 paired lung adenocarcinoma tumor tissues was performed to analyze the protein level of KEAP1. As shown in **Figure 5F**, the protein level of KEAP1 was remarkably downregulated in lung adenocarcinoma tumor tissues, compared to the adjacent non-cancerous tissues. Consistent with the results of immunohistochemistry, the Western blotting for KEAP1 protein in 12 paired lung adenocarcinoma tumor tissues and adjacent normal tissues also confirmed that the protein level of KEAP1 was significantly downregulated (**Figure 5G**). Pearson correlation analysis revealed a significant negative correlation between KEAP1 protein level and miR-141-3p. These results suggested miR-141-3p might regulate the protein level of KEAP1.

To determine whether miR-141-3p could inhibit KEAP1 expression, BEAS-2B cells were transfected with mimics of miR-141-3p to increase the level of miR-141-3p, while A549 and PC9 cells were transfected with inhibitors of miR-141-3p to downregulate the cellular levels of miR-141-3p. As anticipated, mimics of miR-141-3p could inhibit KEAP1 expression, while inhibitors increased KEAP1 levels (**Figure 5H**). KEAP1 is confirmed to bind to NRF2 (nuclear factor erythroid 2–related factor 2) and promote its degradation by the ubiquitin proteasome pathway, which could, in turn, repress the HDAC4 (histone deacetylase 4) expression in LUAD (19). We found that miR-141-3p mimics significantly increased the NRF2 and HDAC4 expressions by repressing the KEAP1 expression, while miR-141-3p inhibitors remarkably suppressed the NRF2 and HDAC4 expression by reliving the suppression of miR-141-3p for KEAP1.

In conclusion, our results confirmed again that miR-141-3p can inhibit KEAP1 by binding to its 3'-UTR.

### circKEAP1 Alleviates the Inhibitory Effect** of miR-141-3p on KEAP1 Expression

**Figure 6:**
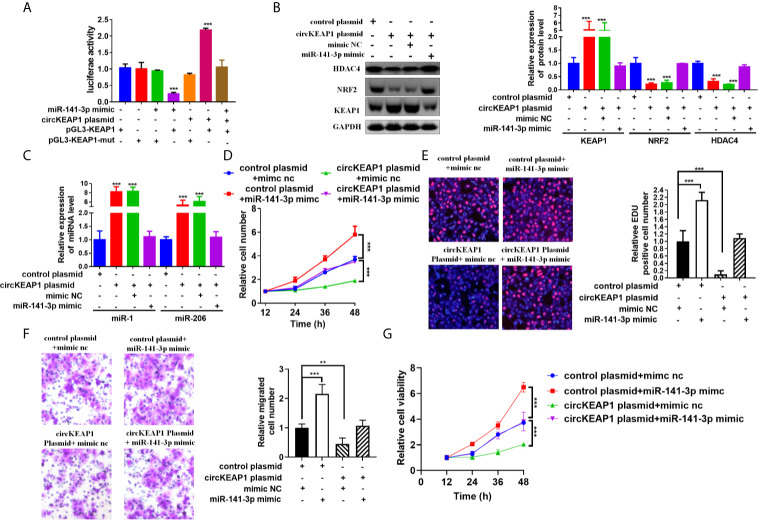
circKEAP1 regulate KEAP1 signaling pathway by sponging miR-141-3p. **(A)** Luciferase activity in A549 cells co-transfected with miRNA mimics or circKEAP1 overepression plasmid and luciferase reporter plasmid which have putative binding site of KEAP1 to miR-141-3p. Luciferase activity was normalized by Renila luciferase activity. **(B)** Western blot analysis of KEAP1, NRF2 and HDAC4 levels in A549 cells co-transfected with mimic nc or miR-141-3p mimic or circKEAP1 overexpression plasmid. **(C)** The expression level of miR-1 and miR-206 in A549 cells co-transfected with mimic nc or miR-141-3p mimic or circKEAP1 overexpression plasmid. **(D)** Cell proliferation analysis for A549 cells co-transfected with mimic nc or miR-141-3p mimic or circKEAP1 overexpression plasmid by CCK-8 assay. **(E)** Cell proliferation analysis for A549 cells co-transfected with mimic nc or miR-141-3p mimic or circKEAP1 overexpression plasmid. **(F)** Cell migration analysis for A549 cells co-transfected with mimic nc or miR-141-3p mimic or circKEAP1 overexpression plasmid by Transwell assay. **(G)** Cell viability analysis for A549 cells co-transfected with mimic nc or miR-141-3p mimic or circKEAP1 overexpression plasmid MTT assay. Data are shown as the means ± standard error of the mean (n = 3), statistical analysis was performed by two-tailed Student’s *t* test; **P < 0.01, ***P < 0.001.

CircRNAs could act as miRNAs sponges to regulate downstream targets (3, 4), by sharing the same miRNAs with mRNA (6, 16). To investigate whether circKEAP1 could relieve the repression of miR-141-3p on KEAP1 expression, the luciferase reporters containing *KEAP1* 3'UTR wild type sequence or muted sequence were co-transfected with circKEAP1 plasmid or miR-141-3p mimic in A549 cells. The results showed that circKEAP1 significantly increased the luciferase activity of KEAP1 wild type reporter, while it did not affect the mutated reporter. Moreover, the increase by the circKEAP1 could be abolished by miR-141-3p overexpression through miR-141-3p mimic (**Figure 6A**). The protein level of KEAP1 was increased in the A549 cells when the circKEAP1 was overexpressed by circKEAP1 plasmid, and resulting in to decrease the NRF2 and HDAC4 expression, but it was rescued by miR-141-3p overexpression (**Figure 6B**). Moreover, the protein level of KEAP1 in the nude mice xenograft model was significantly upregulated in the tumor of circKEAP1 overexpression group, which in turn downregulated the protein level of NRF2 and HDAC4 (Figure S2B). Several studies have confirmed that KEAP1 could suppress the expression of NRF2 and reduce its downstream gene HDAC4 expression, result to increase the tumor suppressor miR-1 and miR-206 (20). In our study, we also found circKEAP1 could significantly increase the expression of miR-1 and miR-206 (**Figure 6C** and **Supplementary Figure S2C**); however, this increase could be attenuated by miR-141-3p overexpression (**Figure 6C**). As the previous studies (21–26), mir-141-3p could promote the proliferation and migration of cancer cells by inhibiting the expression of KEAP1 when the miR-141-3p was overexpressed in the cancer cells by miR-141-3p mimic (**Figures 6D–G**). However, the promotion could be rescued by circKEAP1 overexpression using circKEAP1 plasmid (**Figures 6D–G**).

**Figure 7:**
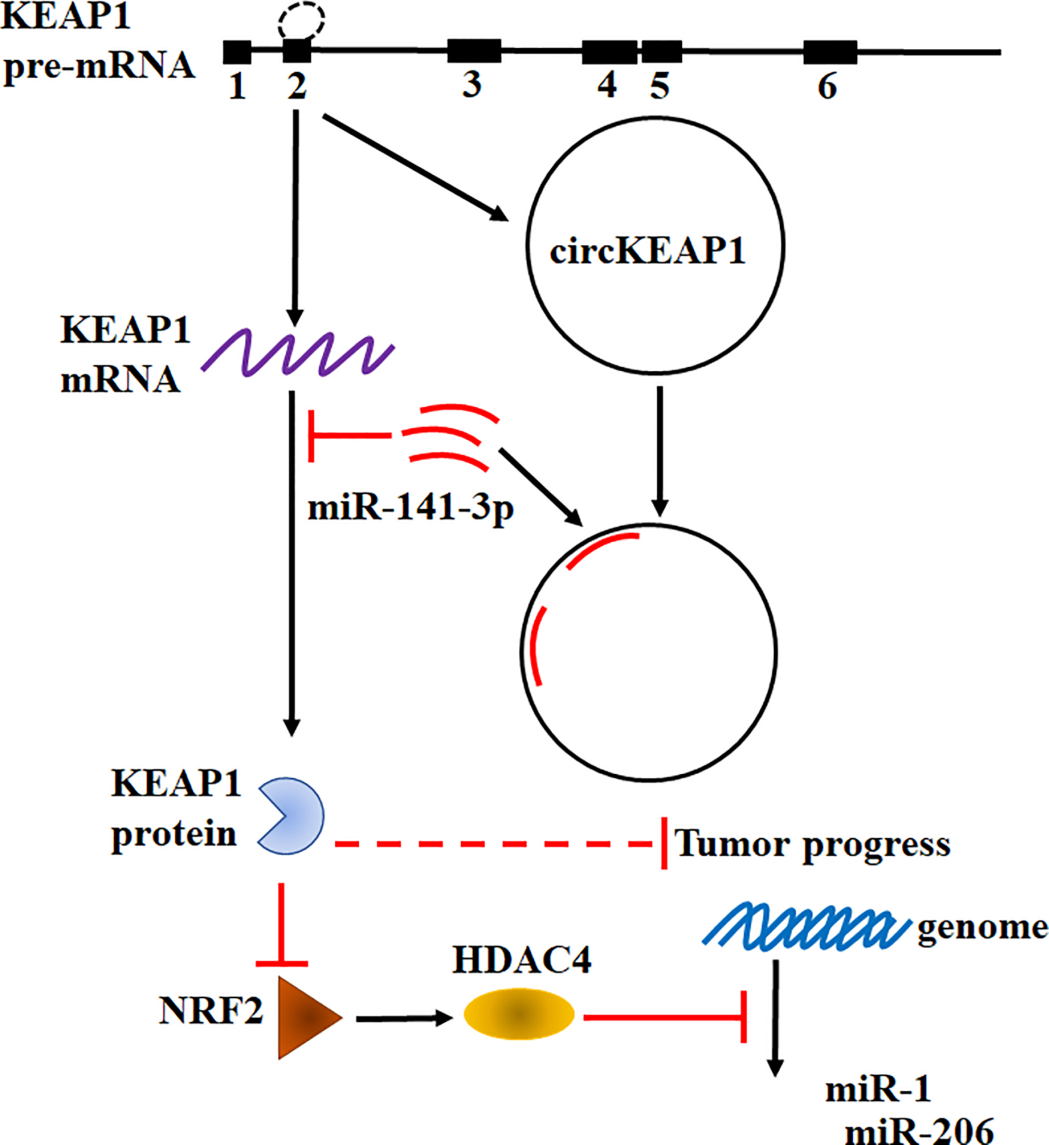
Hypothesis diagram illustrates function and mechanism of circKEAP1 in LUAD progress.

Taken together, our results found circKEAP1 could serve as a sponge for miR-141-3p to regulate KEAP1 and activate the KEAP1/NRF2/HDAC4 signal pathway *via* the ceRNA mechanism to repress tumor growth (**Figure 7**).

## Discussion

Recently, an increasing number of studies have confirmed the dysregulation of circRNAs plays critical roles in modulating tumor development and progression (4). Several circRNA has been revealed to function as oncogenes or tumor suppressors in lung cancer and other types of cancer (4, 27, 28). However, only a few circRNAs have been well characterized. In this study, the profiling of circRNAs in LUAD was obtained by the Human CircRNA microarray. We found circKEAP1 significantly downregulated in LUAD cancer tissues and could suppress the tumor growth. CircKEAP1 exerted its function as a ceRNA that competitively bound to miR-141-3p, then abolished the repression of miR-141-3p for its original gene KEAP1. Elevated KEAP1 could inhibit the expression of NRF2 and HDAC4, and then abolish the suppression of HDAC4 for the tumor suppressor miRNAs miR-1 and miR-206, which result to repress the cell proliferation (**Figure 7**). Our results suggested circKEAP1 could inhibit LUAD cell growth *via* the ceRNA mechanism.

Heterogeneous genetic or epigenetic modifications have been revealed that could modify the oncogenes and tumor suppressor genes expression. The dysregulation of these genes exert their effects on multiple cellular processes in which transient modifications of redox balance might occur, such as cell proliferation. These transient cellular changes are mainly coordinated by KEAP1/NRF2 signaling pathway (19). NRF2 is a transcription factor and could function as a master modulator of cellular defense against toxic and oxidative damage, mitochondrial physiology, differentiation, and stem cell maintenance (29). NRF2 is tightly regulated by KEAP1 (19, 29). In normal cell conditions, KEAP1 forms a ubiquitin ligase complex and targets NRF2 for proteolysis. Upon stress exposure, the KEAP1 releases NRF2 which translocates into the nucleus to form a heterodimeric complex that could recognize the enhancer sequences of antioxidant response elements (AREs) and activates their transcription (19, 29). Since KEAP1 and NRF2 could modulate cell proliferation, it is considered a hallmark in cancer cells of the deregulation of the KEAP1/NRF2 axis. Previous studies have indicated that abnormal states of the KEAP1-NRF2 pathway exist in lung cancer (29, 30). Downregulated KEAP1 has been frequently identified in lung cancer. However, the mechanism remains unknown (30). In this study, we also found KEAP1 was significantly downregulated in LUAD cancer tissues and knockdown of KEAP1 enhance the NRF2 expression. More interestingly, we revealed KEAP1 could encode a circRNA by alternative splicing to sponge miR-141-3p to relive the inhibitor of the miRNA for itself mRNA. In tumor condition, the decrease of the circKEAP1 resulted in the evaluation of miR-141-3p, which in turn suppress the protein level of KEAP1 and increase the NRF2 level. It is reported that KEAP1 could modulate the NRF2 to repress HDAC4 methylated the promoter of tumor suppressor miRNA miR-1 and miR-206 (20). In our study, we found circKEAP1 could upregulate miR-1 and miR-206 by upregulating KEAP1, which could decrease NRF2 expression, and result to suppress the HDAC4 and in turn inhibit the methylation of the promoter of these two miRNAs by HDAC4 (**Figure 7**).

The most significant characteristic of cancer cells is uncontrolled cell proliferation. Previous studies have demonstrated that miR-141-3p was upregulated in multiple human cancers, and high expression of miR-141-3p promoted cancer cell proliferation *via* varying mechanisms (31). For example, Li et al. reported miR-141-3p fostered the growth of cervical cancer cells by targeting FOXA2 (32). Xu et al. found that p53 is directly targeted by miR-141-3p, and miR-141-3p promotes tumor growth through inhibition of p53 pathways (33). Li et al. reported that miR-141-3p could bind with the 3' untranslated region of DAPK1 and repress the expression of DAPK1 which in turn promoted proliferation (34). MTM et al. confirmed miR-141-3p could repress the KEAP1 expression and activate the KEAP1 downstream pathway to modulate cisplatin sensitivity (23). In this study, we found that circKEAP1 could inhibit cell proliferation by acting as a sponge for the miR-141-3p to relieve microRNA repression for the target gene KEAP1.

In conclusion, our study reveals that circKEAP1 competitively binds miR-141-3p to abolish the suppressive effect of miR-141-3p on KEAP1, which results to suppress the NRF2/HDAC4 signal pathway and inhibit tumor progress. Our findings revealed an insight into understanding the KEAP1-NRF2 signal pathway during the development and progression of LUAD, and provide a potential therapeutic approach for LUAD.

## Data Availability Statement

The data sets presented in this study can be found in online repositories. The names of the repository/repositories and accession number(s) can be found in the article/**Supplementary Material**.

## Ethics Statement

The studies involving human participants were reviewed and approved by Harbin Medical University Cancer Hospital. The patients/participants provided their written informed consent to participate in this study. The animal study was reviewed and approved by Harbin Medical University Cancer Hospital.

## Author Contributions

JW designed the experiments. YW, FR, DS, JL, BL, YH, SP, BS, FZ, LY, and YL performed the experiments and analyzed results. SX and JW wrote the manuscript. All authors contributed to the article and approved the submitted version.

## Funding

This work was supported by grants from Special Fund for Scientific and Technological Innovation Talents Research of Harbin Science and Technology Bureau (2017RAQXJ233 to JW), Nn10 Program of Harbin Medical University Cancer Hospital (Nn10pv2017-04 to SX) and 2020 Fundamental Research Funds for the Provincial Universities. (NO.2020-KYYWF-1466)

## Conflict of Interest

The authors declare that the research was conducted in the absence of any commercial or financial relationships that could be construed as a potential conflict of interest.

## Abbreviations

CircRNAs, circular RNAs; LUAD, lung adenocarcinoma; miRNAs, microRNAs; EMT, epithelial–mesenchymal transition; RT-PCR, reverse transcription polymerase chain reaction; qRT-PCR, quantitative reverse transcription polymerase chain reaction; RIP, RNA immunoprecipitation; cDNA, reverse-transcribed RNA; gDNA, genomic DNA; EDU, 5-ethynyl-2'-deoxyuridine; CCK-8, Cell Counting Kit-8; 3'UTR, three prime untranslated region.
