# Supplementary material for: CircKEAP1 Suppresses the Progression of Lung Adenocarcinoma via the miR-141-3p/KEAP1/NRF2 Axis
Source: Front Oncol. 2021 May 31;11:672586. doi: 10.3389/fonc.2021.672586 (PMC8200847; doi:10.3389/fonc.2021.672586)
Supplement: Supplementary file 1 [file Table_1.docx]

**Table s1. Patient characteristics and clinical features for Human CircRNA microarray.**

|  | **Age** | **Gender** | **Tumor subtype** | **Pathological Stage** |
| --- | --- | --- | --- | --- |
| **Case #1** | 60 | Female | Adenocarcinoma | II A |
| **Case #2** | 58 | Male | Adenocarcinoma | I B |
| **Case #3** | 67 | Female | Adenocarcinoma | III A |
| **Case #4** | 70 | Male | Adenocarcinoma | I B |
| **Case #5** | 70 | Male | Adenocarcinoma | I |
| **Case #6** | 59 | Male | Adenocarcinoma | III A |
| **Case #7** | 59 | Female | Adenocarcinoma | III A |
