# Supplementary material for: CircKEAP1 Suppresses the Progression of Lung Adenocarcinoma via the miR-141-3p/KEAP1/NRF2 Axis
Source: Front Oncol. 2021 May 31;11:672586. doi: 10.3389/fonc.2021.672586 (PMC8200847; doi:10.3389/fonc.2021.672586)
Supplement: Supplementary file 2 [file Table_2.docx]

**Table s2. Patient characteristics and clinical features for validation of circKEAP1.**

|  |  | **LUAD (n=105)** |
| --- | --- | --- |
| **Average age (years)** |  | **66.3±9.8** |
| **Gender** | **Female** | **49** |
|  | **Male** | **56** |
| **Stage** | **Ⅰ** | **47** |
|  | **Ⅱ** | **32** |
|  | **III** | **16** |
|  | **IV** | **10** |
