# Supplementary material for: CircKEAP1 Suppresses the Progression of Lung Adenocarcinoma via the miR-141-3p/KEAP1/NRF2 Axis
Source: Front Oncol. 2021 May 31;11:672586. doi: 10.3389/fonc.2021.672586 (PMC8200847; doi:10.3389/fonc.2021.672586)
Supplement: Supplementary file 3 [file Table_3.docx]

**Table s3. the primers used for qRT-PCR.**

| **Gene** | **Forward** | **Reverse** |
| --- | --- | --- |
| circKEAP1 | GCATTTTGGGGAGGTCCCTG | TGCGGTTGCCATGCTGGGAGGGCG |
| KEAP1 | GTGTCCATTGAGGGTATCCACC | GCTCAGCGAAGTTGGCGAT |
| GAPDH | GCCTTCCGTGTCCCCACTGC | CAATGCCAGCCCCAGCGTCA |
