# Supplementary material for: CircKEAP1 Suppresses the Progression of Lung Adenocarcinoma via the miR-141-3p/KEAP1/NRF2 Axis
Source: Front Oncol. 2021 May 31;11:672586. doi: 10.3389/fonc.2021.672586 (PMC8200847; doi:10.3389/fonc.2021.672586)
Supplement: Supplementary file 5 [file Table_5.docx]

**Table s5. The correlation between circKEAP1 expression and clinicopathological features in LUAD patients.**

| **Clinical parameter** | **circKEAP1** | **circKEAP1** | **Chi-squared test P value** |
| --- | --- | --- | --- |
|  | **High no. cases** | **Low no. cases** |  |
| Age (years) |  |  | >0.05 |
| <60 | 20 | 30 |  |
| >60 | 22 | 33 |  |
| Gender |  |  | >0.05 |
| Male | 16 | 21 |  |
| Female | 14 | 10 |  |
| TNM stage |  |  | <0.05* |
| I-II | 53 | 26 |  |
| III-IV | 9 | 17 |  |
| Lymphatic metastasis |  |  | <0.05* |
| Yes | 22 | 36 |  |
| No | 30 | 17 |  |
