# Supplementary material for: CircKEAP1 Suppresses the Progression of Lung Adenocarcinoma via the miR-141-3p/KEAP1/NRF2 Axis
Source: Front Oncol. 2021 May 31;11:672586. doi: 10.3389/fonc.2021.672586 (PMC8200847; doi:10.3389/fonc.2021.672586)

**Supplement tables and figures**

**Table s1. Patient characteristics and clinical features for Human CircRNA microarray.**

**Table s2. Patient characteristics and clinical features for validation of circKEAP1.**

**Table s3. the primers used for qRT-PCR.**

**Table s4. The dysregulated circRNAs in seven paired samples of lung adenocarcinoma tissues and adjacent normal tissues by the human circRNA microarray. (Fold change>2, p<0.05)**

**Table s5. The correlation between circKEAP1 expression and clinicopathological features in LUAD patients.**

**Table s6. The dysregulated miRNAs in seven paired samples of LUAD and adjacent normal tissues was analyzed by Human miRNA Expression Assay. (Fold change>2, p<0.05)**

**Table s7. the potential binding sites of miRNAs in the circKEAP1 by Targetscan.**

**Figure S1. Expression levels of miR-141-3p in A549 cells transfected with mimic nc or miR-141-3p mimic. Data are shown as the means ± standard error of the mean (n =3), statistical analysis was performed by two-tailed Student’s t-test; *P < 0.05, **P < 0.01, ***P < 0.001.**


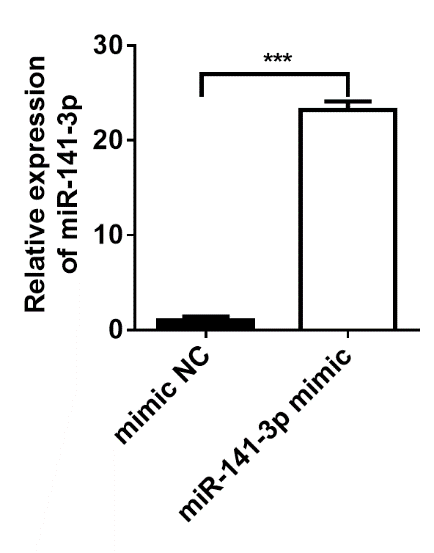


**Figure S2. circKEAP1 relieved repression of miR-141-3p for KEAP1 expression. (A)** Expression levels of miR-141-3p in xenografted tumors. **(B)** Western blot analysis of KEAP1, NRF2 and HDAC4 levels in xenografted tumors. **(C)** Expression levels of miR-1 and miR-206 in xenografted tumors. Data are shown as the means ± standard error of the mean (n =3), statistical analysis was performed by two-tailed Student’s t-test; *P < 0.05, **P < 0.01, ***P < 0.001.


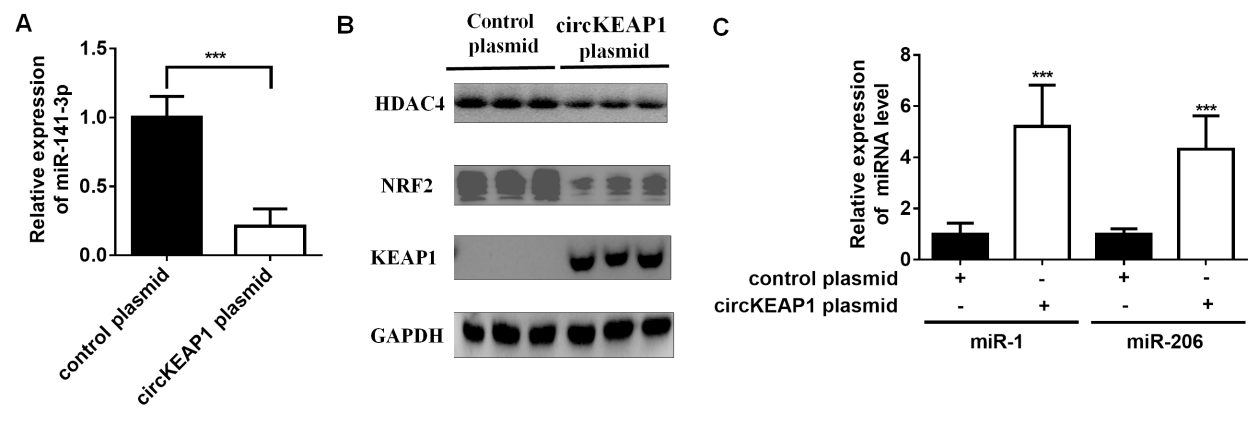

Supplement: Supplementary file 8 [file DataSheet_1.docx]
